# Laparoscopic Versus Open Surgery for Advanced-Stage Ovarian Cancer: A 10-Year Data Analysis

**DOI:** 10.7759/cureus.106112

**Published:** 2026-03-30

**Authors:** Kheyal Khalil, Maria Habib, Anam Riaz, Shahid Khattak, Aamir Syed

**Affiliations:** 1 Surgical Oncology, Shaukat Khanum Memorial Cancer Hospital and Research Centre, Lahore, PAK; 2 Gynaecologic Oncology, Shaukat Khanum Memorial Cancer Hospital and Research Centre, Lahore, PAK

**Keywords:** advanced ovarian cancer, gynecological cancers, laparoscopic surgery, minimally invasive surgery, ovarian cancer surgery

## Abstract

Objective

The study’s objective is to compare long- and short-term surgical outcomes as well as progression-free and overall survival in patients with laparoscopic versus open surgery for advanced-stage ovarian cancer.

Methods

A total of 171 patients with advanced stage III/IV ovarian cancer who underwent ovarian cancer surgery were included in this retrospective cohort study. The surgeries performed from January 2014 to January 2024 were reviewed. Patient characteristics, such as age, BMI, cancer histology and stage, and treatment received (chemotherapy and surgery), will be noted. The study compares surgical outcomes as well as overall survival and progression-free survival (PFS) in patients.

Results

The mean age of this study cohort was 53.47 ± 11 years (range 27-87 years) with an average BMI of 27.7 ± 5.8. 17 (10%) patients had total laparoscopic surgery, whereas 154 (90%) patients had open surgery with a midline incision. Interestingly, patients receiving an extra cycle of chemotherapy pre-operatively were found to have undergone laparoscopic surgery (p = 0.009). The average blood loss for the laparoscopy group was 82 ± 50 mL, whereas with open surgery, it was 164 ± 125 mL. On the other hand, the average length of surgery was longer for laparoscopy (231 ± 94 minutes) as compared to open surgery. The length of hospital stay was shorter for the laparoscopy group (3.24 ± 1.2 days). The overall and PFS were longer in the open surgery group, but this difference was not statistically significant.

Conclusion

Laparoscopy offers short-term benefit in terms of intraoperative and postoperative indicators, but no significant survival benefit was noted in one group over the other.

## Introduction

Ovarian cancer is one of the most common gynecological malignancies [1]. The treatment for advanced-stage ovarian cancer is multimodal, including neoadjuvant chemotherapy and debulking surgery [2]. Over the years, surgical techniques have been modified to improve surgical outcomes, and with the advent of the laparoscopic approach, the postoperative course has been completely transformed [3]. While the efficacy of minimally invasive surgery has been established since its introduction, there is a question of its usefulness in achieving complete gross resection (CGR) in oncological surgeries. The role of complete resection is fundamental in the treatment of ovarian cancer, with a definitive role of optimal debulking on progression-free and overall survival rates [4]. In advanced-stage ovarian cancers, owing to the anatomical location of the tumor and the pathophysiology of its spread, oncological ovarian surgeries can be technically quite complex [5]. The extent of these surgeries includes operating on colorectal and urological structures and diffuse peritoneal disease in advanced-stage ovarian cancer that may not be amenable to CGR either laparoscopically or sometimes at all. There is a school of thought that open surgery may provide the additional benefit of tactile feedback lost in the laparoscopic approach to resect widespread peritoneal disease [6].

This benefit, however, needs to be weighed against the potential drawbacks associated with a bigger surgical incision on the postoperative course. In addition to the immediate postoperative course, it may be of value to note the effect of surgical technique on long-term effects on progression-free and overall survival that would reflect on the quality of optimal cytoreduction achieved with these two surgical techniques [7].

Another argument relevant to the skepticism surrounding laparoscopic surgery is a result of the well-known Laparoscopic Approach to Cervical Cancer (LACC) trial [8]. This was a phase III, non-inferiority randomized trial that compared minimally invasive radical hysterectomy to open surgery for stages 1A1, 1A2, and 1B1 cervical cancer. It is of note that minimally invasive surgery in this trial included robotic surgery. The conclusion of this trial showed a worse disease-free survival and overall survival in patients who underwent minimally invasive hysterectomy as compared to open surgery. This conclusion led to the recommendation of open radical hysterectomy as the standard of care for early-stage cervical cancer. The LACC trial opened the debate regarding surgical technique for other gynecological malignancies, especially advanced ovarian cancer, which can be quite a challenging surgery for optimal debulking.

Our study was conducted at a high-volume center for ovarian cancer surgery, where we have many open and laparoscopic surgeries. This study did not include robotic surgery. The approach considerations included surgical expertise, extent of disease, and safe surgical technique, among others [9]. We compared short-term surgical outcomes and long-term survival outcomes to ascertain if there was any significant difference that could influence our current practice.

This article was previously presented as a meeting abstract at the 2025 AANS Shalamar IRC RCOG summit on January 15, 2025.

## Materials and methods

A total of 171 patients with advanced stage III/IV ovarian cancer who underwent ovarian cancer surgery were included in this retrospective cohort study. The study cohort was divided into two groups: 17 (10%) patients underwent laparoscopic surgery, and 154 (90%) patients underwent open surgery. The surgeries performed from January 2014 to January 2024 were reviewed.

Patient characteristics, such as age, BMI, cancer histology and stage, and treatment received (chemotherapy and surgery), were noted. Patients with early-stage ovarian cancer (stage I and stage II disease), BMI > 40, women who did not undergo surgery for ovarian cancer, patients with more than one primary cancer, inadequate chemotherapy data, and patients who were lost to follow-up were excluded from the study cohort. Patients with Eastern Cooperative Oncology Group (ECOG) performance status [10] of >3 and those undergoing surgery for recurrent and/or residual disease were also excluded from this study. The reasoning behind this is that patients who had a poor performance status at presentation were inherently not comparable to the rest of the group. Similarly, patients who were undergoing surgery for recurrent and/or residual disease had a technically difficult procedure when compared to patients having their first debulking operation, and hence could not be compared as surgical technique and complexity had a direct impact on the variables being studied.

**Figure 1:**
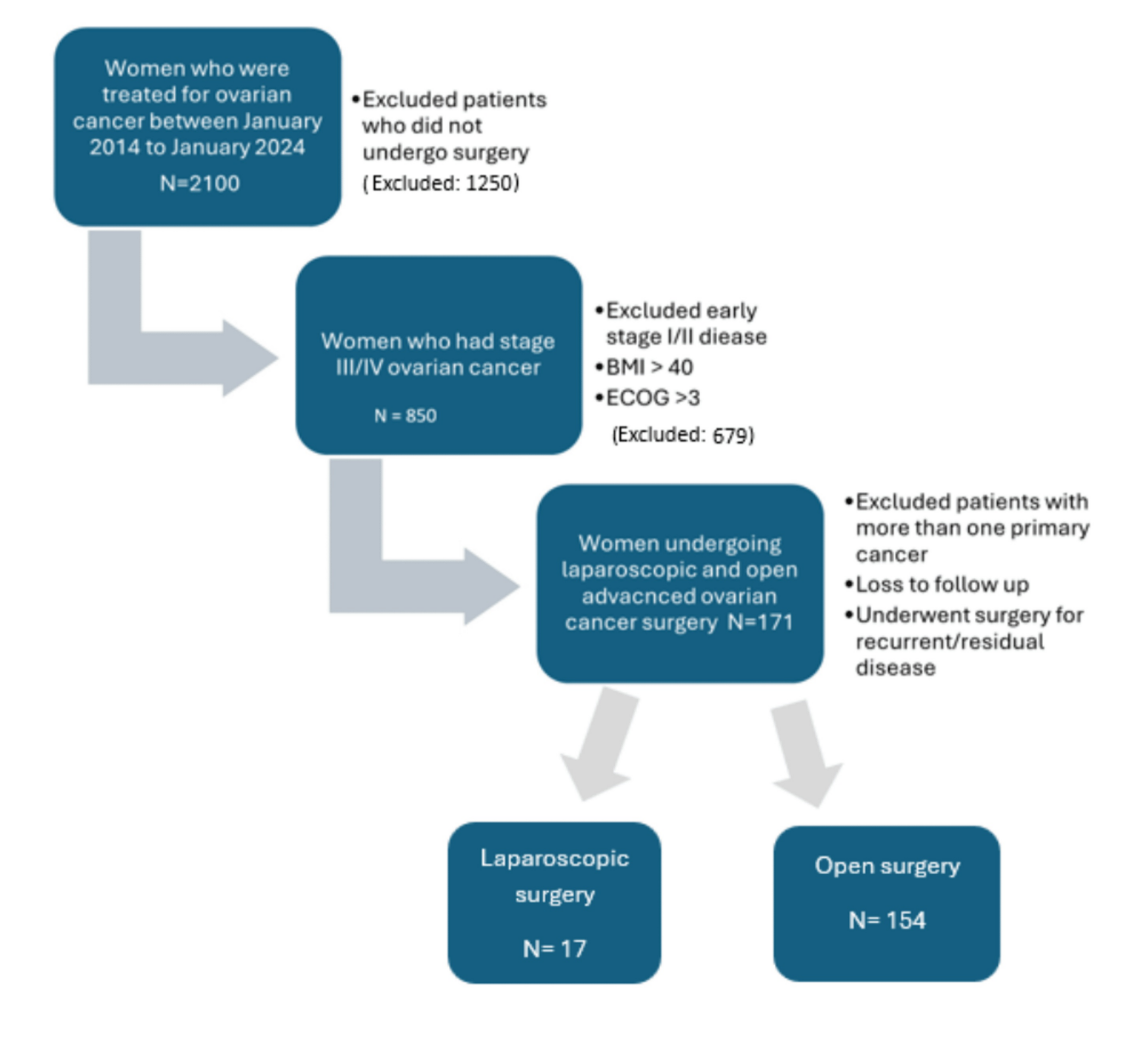
Inclusion and exclusion criteria Image was created using Microsoft Word (Microsoft® Corp., Redmond, WA).

The patients were selected for laparoscopic surgery if no radiological evidence was present that could preclude this surgical technique, such as huge masses requiring a big incision. The rest of the patients were started off with a diagnostic laparoscopy to establish resectability and feasibility to proceed laparoscopically. If proceeding with laparoscopy, they were included in the laparoscopy group; however, if proceeding with laparotomy, they were included in the open surgery group. The entire study cohort had CGR achieved, the surgery included total abdominal hysterectomy, bilateral salpingo-oophorectomy, infracolic omentectomy for all the patients, with some undergoing appendectomy, bowel resection, anastomosis, pelvic lymphadenectomy, cholecystectomy, and splenectomy in individual cases as required. The study compared short-term surgical outcomes such as blood loss volume, time spent in hospital, incidence of postoperative fever, ICU admission, length of surgery, number of neoadjuvant chemotherapy cycles, and rate of blood transfusion, as well as overall survival and progression-free survival (PFS) in patients (Figure 1).

Demographic details for both patient groups, including age, BMI, ECOG performance status, and CA 125 levels, were also compared to ensure there was no confounding bias between the two cohorts. All patients who were included underwent optimal debulking surgery irrespective of the surgical technique. The patient selection for undergoing surgical versus laparoscopic surgery included using an initial laparoscopic survey on all patients to determine resectability and the feasibility of using laparoscopy for debulking disease in the respective patients. Other factors included surgeon’s prerogative in deciding surgical technique, owing to patient factors such as the presence of large ovarian masses not amenable to the minimally invasive approach. Care was taken to minimize the chances of upstaging disease by incorrect surgical approach; laparoscopic surgery was attempted after ensuring that it could be safely performed. Some procedures had to be converted from laparoscopy to open procedure intraoperatively, and these were included in the open surgery group.

The immediate surgical outcomes, including the amount of blood loss, rate of blood transfusion, length of hospital stay, postoperative admissions to the ICU, number of neoadjuvant chemotherapy cycles, length of surgery, and incidence of postoperative fever, were noted. The long-term outcomes included progression-free and overall survivals since the date of the surgery till the date of last follow-up and/or the date of death. 

The data were entered and analyzed using SPSS (IBM SPSS Statistics for Windows, IBM Corp., Version 27.0, Armonk, NY). Descriptive analysis was performed on all variables. For categorical variables, frequencies and percentages were calculated, while for continuous variables, the mean (SD) or median (range) was calculated depending on the distribution of the data. The distribution of data was assessed using the Kolmogorov-Smirnov test of normality. For the comparison of categorical variables, the chi-square test, Fisher’s exact test, or the likelihood ratio test was applied. For the comparison of continuous variables based on groups, the Mann-Whitney U test or t-test was used, depending on the data distribution. For overall and relapse-free survival, Kaplan-Meier analysis was performed, and for group-based comparisons, the log-rank test (Mantel-Cox test) was applied. Yearly survival rates were also calculated along with their respective 95% confidence intervals through statistical computing language R (R Foundation for Statistical Computing, Vienna, Austria). A p-value of less than 0.05 was considered significant.

## Results

The mean age of this study cohort was 53.47 ± 11 years (range 27-87 years), 53.62 ± 11.1 years for the open surgery group, and 52.06 ± 11 years in the laparoscopy group (p > 0.05). The average BMI was 25.9 ± 5.95 and 27.9 ± 5.79 for the laparoscopy versus open groups, respectively (p > 0.05). The ECOG performance status was 0 for more than half of the study cohort (96, 56.1%) and a score of 1 for 68 (39.8%) of the patients, and scores of 2 and 3 for less than 5% (N = 7) of patients (p > 0.05). None of these differences was statistically significant, and hence the two groups were comparable (Table 1).

**Table 1:** Demographic findings

	Laparoscopy	Open surgery	N (171)	p-value
Age in years (mean ± SD)	52.06 ± 11.1	53.62 ± 11.1	53.47 ± 11	0.582
BMI (mean ± SD)	25.9 ± 5.95	27.9 ± 5.79	27.7 ± 5.82	0.169
ECOG performance status - 0	9 (52.9%)	87 (56.4%)	96 (56.1%)	0.787
ECOG performance status - 1	8 (47.1%)	60 (38.9%)	68 (39.8%)	-
ECOG performance status - 2	0	4 (2.6%)	4 (2.3%)	-
ECOG performance status - 3	0	3 (2.1%)	3 (1.8%)	-

The most common histology was high-grade serous carcinoma (158, 92.4%), followed by low-grade serous carcinoma (four, 2.3%), endometrioid carcinoma (four, 2.3%), mucinous carcinoma (one, 0.6%), and clear cell carcinoma (four, 2.3%) of the ovary. A total of 139 (81.3%) patients had stage III disease, whereas 32 (18.7%) had stage IV disease. The majority of patients were staged radiologically (145, 84.8%) upon diagnosis using CT scans for chest, abdomen, and pelvis; 21 (12.3%) were staged with laparoscopic assessment, and five (2.9%) patients required both.

The study cohort received the standard carboplatin/paclitaxel chemotherapy regimen, with some patients receiving additional bevacizumab. Only six (3.5%) patients received a PARP inhibitor.

The patients underwent total hysterectomy (157, 91.8%), bilateral salpingo-oophorectomy (167, 97.7%) and infracolic omentectomy (157, 91.8%) at the minimum with additional procedures such as appendectomy (16, 9.4%), bowel resection (21, 12.3%), stoma placement (nine, 5.3%), pelvic lymphadenectomy (16, 9.4%), depersonalization (three, 1.8%) in a select few cases.

A total of 17 (10%) patients had total laparoscopic surgery, whereas 154 (90%) patients had open surgery with a midline incision. The patients who underwent laparoscopic surgery had lesser blood loss (82.35 ± 50 mL versus 164.22 ± 125 mL; p < 0.05) and shorter hospital stay (3.24 ± 1.3 days versus 4.53 ± 1.4 days; p < 0.05). On the other hand, the average length of surgery was longer for laparoscopy (231.65 ± 94 minutes) as compared to open surgery (167.55 ± 65 minutes; p < 0.05). This was an anticipated result owing to this center being a teaching institute for laparoscopic surgery, and hence, a longer operation time was expected for minimally invasive surgeries. The incidence of ICU admission, postoperative fever, and postoperative blood transfusion was low for the entire study cohort; therefore, bivariate analysis of these results did not reach statistical significance (Table 2).

**Table 2:** Postoperative outcomes

	Open surgery	Laparoscopic surgery	p-value
Estimated blood loss (mL)	164.22 ± 125	82.35 ± 50	0.001
Blood transfusion	21.4%	5.9%	0.20
Hospital stay (days)	4.53 ± 1.4	3.24 ± 1.3	<0.001
ICU admission	5.2%	0%	1.00
Neoadjuvant chemo cycles (number)	5.40 ± 1.6	6.54 ± 1.7	0.003
Length of surgery (minutes)	167.55 ± 65	231.65 ± 94	0.004
Postoperative fever	3.2%	5.9%	0.47

**Figure 2:**
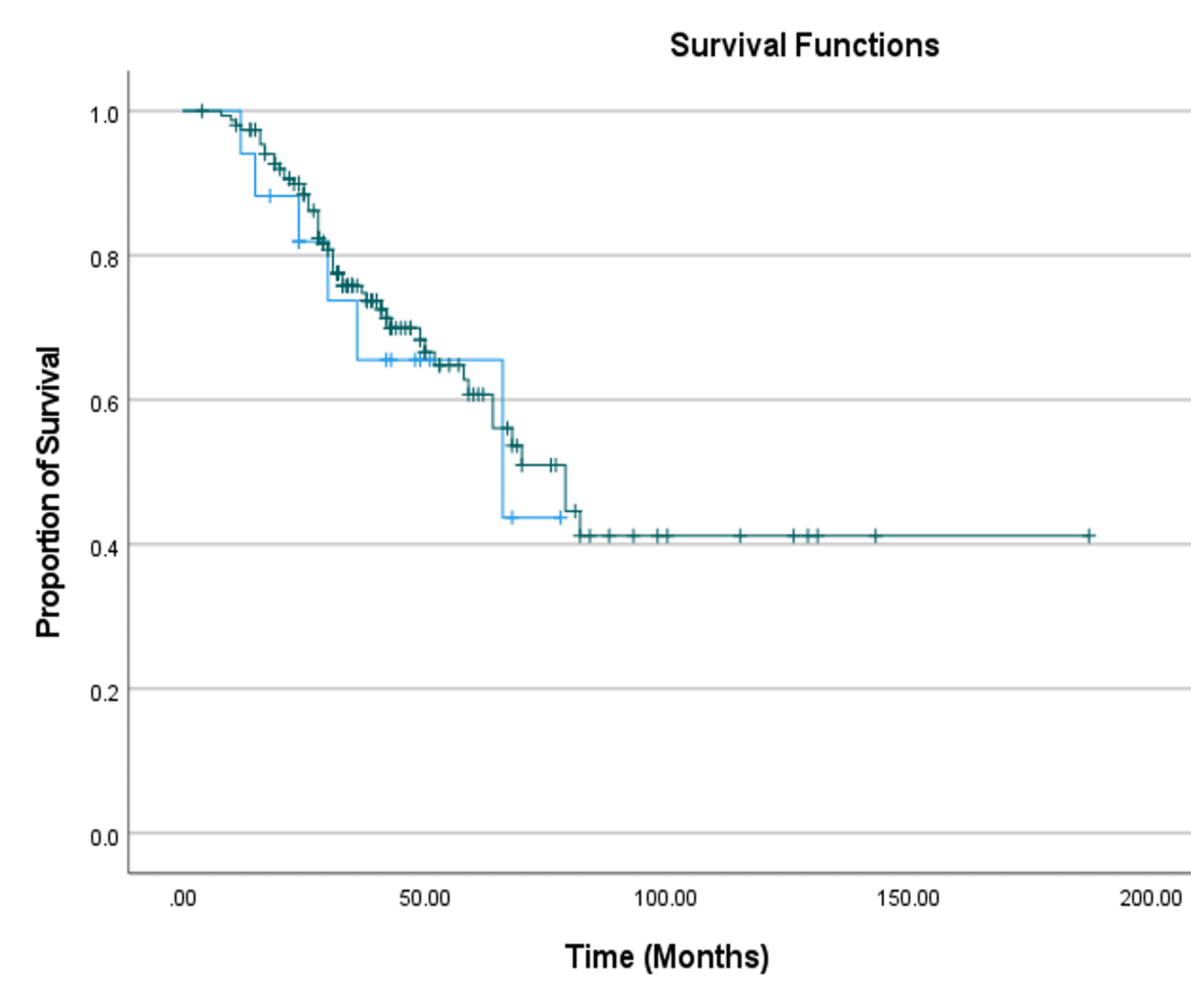
Overall survival Kaplan-Meier (K-M) curve Laparoscopy - blue line. Open - green line. Image was generated using SPSS (IBM SPSS Statistics for Windows, IBM Corp., Version 27.0, Armonk, NY).

**Figure 3:**
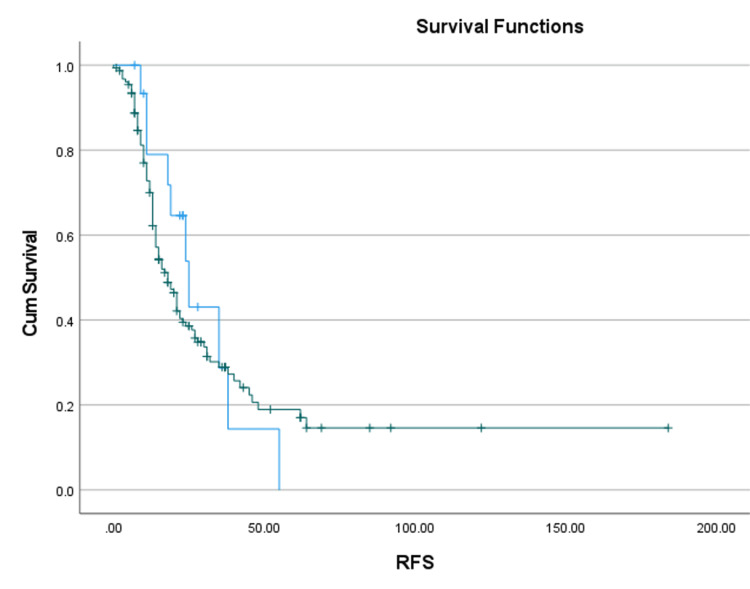
Recurrence-free survival (RFS) Kaplan-Meier (K-M) curves Laparoscopy - blue line. Open - green line. Image was generated using SPSS (IBM SPSS Statistics for Windows, IBM Corp., Version 27.0, Armonk, NY).

Interestingly, patients receiving an extra cycle of chemotherapy pre-operatively were found to have undergone laparoscopic surgery (p = 0.009). This result provided an insight into the possibility of considering an extra cycle of chemotherapy before interval debulking surgery, which might actually increase the chances of achieving a minimally invasive procedure. The overall and PFSs were longer in the open surgery group (104.4 ± 9.2 months and 44.5 ± 6.5 months, respectively) as compared to the laparoscopy group, but this difference was not statistically significant (Figures 2-3).

There were six (35%) deaths recorded in the laparoscopy group and 50 (32.5%) in the open surgery group of patients. A recurrence-free survival rate of four years was recorded, as laparoscopy for advanced ovarian cancer has been relatively recently adopted in routine practice (Table 3).

**Table 3:** Survival outcomes

Surgery	Overall survival		Recurrence-free survival	
	Event (%)	Five-year survival rate (95% CI)	Event (%)	Four-year survival rate (95% CI)
Open	50 (32.5%)	60.8 % (51.1-72.2)	102 (66.23%)	18.9% (12.1-29.7)
Laparoscopy	6 (35.3%)	65.5% (44.7-96.2)	11 (64.71%)	13.5% (2.4-77.4)

## Discussion

This retrospective cohort study clearly demonstrated previously established better short-term perioperative and postoperative outcomes of laparoscopic surgery. The survival analysis in terms of recurrence-free and overall survivals also did not demonstrate benefit when comparing open versus laparoscopic surgery.

A systematic review and meta-analysis done in 2024 on minimally invasive surgery for the management of ovarian cancer highlighted that minimally invasive surgery did offer perioperative benefit and may be used in selective patients. Also, in the early stage of the disease, there was no difference in survival between laparotomy and minimally invasive surgery. The rate of optimal debulking in advanced-stage ovarian cancer was comparable to that of early-stage disease. However, this analysis has a potential bias due to the inclusion of observational studies [11].

Another systematic review performed in 2019 that considered laparoscopy versus laparotomy for advanced ovarian cancer demonstrated that laparoscopy can be used to establish resectability, followed by careful selection of patients for appropriate and adequate surgical resection of disease. The survival outcomes are comparable to those of patients who had laparotomy; the emphasis, however, remained on patient selection [12]. This has been similar to findings from our local study as well.

A propensity-matched comparison performed between laparoscopy and traditional surgery for epithelial ovarian cancer showed that while midline laparotomy is the gold standard, especially for supra-mesocolic disease, laparoscopy is an effective alternative in patients whose disease is amenable to CGR with minimal residual tumor. This study also reiterated the similar theme of cautious patient selection [13].

Another systematic review looked at choosing laparotomy or a minimally invasive technique for recurrent ovarian cancer surgery. The studies included in this analysis also had patients who underwent robotic-assisted surgery and patients who had laparoscopic surgery. In their analysis, it was noted that patients chosen for a minimally invasive secondary surgery were usually the ones with a localized disease site as opposed to widespread disease. There was a similar emphasis on patient selection as the most important predictor of a successful surgical outcome. However, it is pertinent to note that a specific criterion for this patient selection was not found [14].

A 2022 study conducted using retrospective data from a single oncological center that compared laparoscopy to open debulking surgery had similar results of improved short-term perioperative outcomes in terms of blood loss volume, complication, and ICU admission rate, with no difference in survival outcomes. The inverse probability of treatment weighting propensity score method was applied to improve discrepancies in other variables, thus making the analysis more robust. There was a suggestion of performing randomized controlled trials for further evaluation [15].

Another retrospective cohort study included patients with stage III ovarian cancer from January 2011 to March 2019 who underwent laparoscopy versus laparotomy for primary ovarian cancer surgery. They concluded that after careful patient selection, the outcomes of both surgical techniques were similar in terms of post-surgical and oncological outcomes. A recommendation for prospective randomized trials was made as well to evaluate overall oncological outcomes [9].

A recent review published in 2023 made a summative statement regarding the ongoing discussion regarding the mode of surgery for ovarian cancer. This review acknowledges the conclusion of various studies that propose benefits of minimally invasive surgical techniques in the immediate postoperative period and surgical outcomes, while offering similar survival benefits; however, they propose that this might be an oversimplification. The technical difficulties with minimally invasive surgery undermine the use of this data, as complete resection is sometimes not possible without a laparotomy [16]. This has also been acknowledged in several studies that patient selection is key to benefit from the minimally invasive technique.

That leads to the ongoing phase III non-inferiority randomized clinical trial, Laparoscopic Cytoreduction After Neoadjuvant Chemotherapy (LANCE), which will address this very question and help formulate what would be the expected standard of care for advanced ovarian cancer surgery in the near future [17].

## Conclusions

Laparoscopy offers short-term benefit in terms of intraoperative and postoperative indicators, but no significant survival benefit was noted in one group over the other. The key is careful patient selection for deciding the mode of surgery. The prospective ongoing randomized trial is a step in the right direction to address this debate.
